# Reproductive stoppage in autism spectrum disorder in a population of 2.5 million individuals

**DOI:** 10.1186/s13229-019-0300-6

**Published:** 2019-12-11

**Authors:** Ralf Kuja-Halkola, Henrik Larsson, Sebastian Lundström, Sven Sandin, Azadeh Chizarifard, Sven Bölte, Paul Lichtenstein, Emma Frans

**Affiliations:** 1 Department of Medical Epidemiology and Biostatistics, Karolinska Institutet, PO Box 281, SE-171 77 Stockholm, Sweden; 2 School of Medical Sciences, Örebro University, Örebro, Sweden; 3 Gillberg Neuropsychiatry Centre; Centre for Ethics, Law and Mental Health, University of Gothenburg, Gothenburg, Sweden; 4 Department of Psychiatry, Icahn School of Medicine at Mount Sinai, New York, USA; 5 Seaver Autism Center for Research and Treatment at Mount Sinai, New York, USA; 6 Department of Statistics, Stockholm University, Stockholm, Sweden; 7 Center of Neurodevelopmental Disorders (KIND), Centre for Psychiatry Research; Department of Women’s and Children’s Health, Karolinska Institutet & Stockholm Health Care Services, Region Stockholm, Stockholm, Sweden; 8 Child and Adolescent Psychiatry, Stockholm Health Care Services, Region Stockholm, Stockholm, Sweden; 9 Curtin Autism Research Group, School of Occupational Therapy, Social Work and Speech Pathology, Curtin University, Perth, WA Australia

**Keywords:** Autism, Autism spectrum disorder, Reproduction, Reproductive stoppage, Fecundity

## Abstract

**Background:**

It has been suggested that parents of children with autism spectrum disorder (ASD) curtail their reproduction, a phenomenon known as reproductive stoppage. To investigate the presence of reproductive stoppage, we followed the reproduction in mothers of children with or without an ASD diagnosis using Swedish population-based registries.

**Methods:**

We followed all families with first child born in 1987 or later. In total 2,521,103 children, nested within 1,270,017 mothers, were included. Exposure was presence of ASD diagnosis in earlier born siblings, and outcome was considered as (1) inter-pregnancy interval and (2) number of subsequent children.

**Results:**

Analyses of inter-pregnancy intervals showed that the association differed across birth orders, with a lower rate of second children when first child had ASD diagnosis, but an increased rate of third and higher birth orders in families where a previous child had an ASD diagnosis. When all birth orders were simultaneously considered, families with a child with an ASD diagnosis were less likely to have another child (hazard ratio (HR), 0.79; 95% confidence interval [95% CI], 0.78–0.80). However, when adjusted for birth order, the association was close to null (HR, 0.97; 95% CI, 0.96–0.99), and after additional adjustments (maternal age, birth period, sex, paternal age, and maternal education), the association disappeared (HR, 1.00; 95% CI, 0.99–1.02). In analyses of subsequent children, after adjustment for covariates, families with an ASD diagnosis had 4% more subsequent children (rate ratio, 1.04; 95% CI, 1.03–1.05).

**Limitations:**

The study was undertaken in a country with largely tax-funded healthcare; results may not generalize to other societies. Following the current dominating umbrella concept of ASD, we did not differentiate between the ASD sub-diagnoses; it is possible that reproductive patterns can be dependent on ASD subtypes and the severity and composition of ASD phenotypes and comorbidities.

**Conclusions:**

This study does not support a universal reproductive stoppage effect in ASD families, when birth order and other factors are considered. Therefore, proper attention to birth order and other factors may alleviate potential bias in familial aggregation studies of ASD.

## Background

It has been suggested that parents who have a child with autism spectrum disorder (ASD) may curtail their reproduction compared to parents who do not have a child with these impairments. This phenomenon is often referred to as reproductive stoppage [1–4]. The definition of reproductive stoppage varies and has in recent studies been operationalized as a change in inter-pregnancy interval (time to next child) depending on whether a prior born child has an ASD diagnosis [3, 4]. However, a change in total number of subsequent children is equally compatible with the concept, as suggested in a recent study by Wood et al., who define reproductive stoppage as “following the diagnosis of ASD in a family, parents may change plans regarding their family size” [5].

Stoppage is often mentioned as a potential source of bias in family studies of ASD [3, 4, 6–9]. Recent research found evidence of reproductive stoppage when estimating inter-pregnancy intervals or number of second-born children in parents whose first-born had ASD [3, 4]. Still, to the best of our knowledge, neither an overall effect on inter-pregnancy intervals—estimating a combined effect over different birth orders—nor the effect on number of subsequent children, as an estimate of stoppage on total family size, has been investigated in a population-based cohort. It therefore remains unknown whether evidence of reproductive stoppage remains if (i) combined over birth orders and if (ii) it is present when assessing number of subsequent children. Considering its potential to introduce bias in studies on ASD, it is of great importance to scrutinize the potential stoppage effect, not in specific subsamples but in the population as a whole.

The aim of this study was to examine reproductive stoppage in a total population followed up longitudinally, including all births in Sweden between 1987 and 2013. We examined the effect of having a child with ASD diagnosis on mother’s reproduction. More specifically, compared to families without children with ASD diagnosis, we investigated both the inter-pregnancy intervals and the number of subsequent children in families having:

1. Any earlier born children with an ASD diagnosis using all birth orders combined,
2. Immediately previous born child with an ASD diagnosis for all birth orders combined,
3. Different combinations of prior born children with ASD diagnoses.

## Methods

### Study cohort

We identified the study cohort by linking population-wide registers in Sweden. From the Total Population Register [10], we identified all individuals born between January 1, 1987, and December 31, 2013, (*N* = 3,268,205). The Multi-Generation Register [11] was used to identify parents and birth order by mother, excluding individuals without identifiable parents (133,253 excluded). We included only births of mothers who had their first child in 1987 or later (381,349 excluded). We excluded families with multiple births (e.g., twins; 113,552 excluded) and only included the first five live born children by mother in each family (10,893 excluded), ignoring whether the siblings were full or half. Mothers in these families were followed until death, date of first emigration, or end of follow-up (December 31, 2013), whichever came first. Children born after the mothers were censored due to emigration out of Sweden were excluded (108,055 excluded) resulting in a dataset consisting of 2,521,103 births nested within 1,270,017 mothers. For all analyses, we further limited the analytic dataset to births prior January 1, 2012 (to ensure a possibility of at least 2 years of follow-up), resulting in 2,296,137 inter-pregnancy intervals. Of these, 1,246,227 ended in another birth and 1,049,910 were censored.

This study was conducted on anonymized data and the study was approved by the Regional Ethical Review Board in Stockholm, Sweden.

### Exposures

We linked the study cohort dataset to the National Patient Register, which gathers diagnoses from inpatient healthcare throughout the follow-up, and from outpatient visits to specialist care from 2001 and onwards [12], and identified ASD diagnoses in the children according to International Classification of Diseases, 9th revision, (ICD-9; years 1987–1996, code 299) and ICD, 10th version (ICD-10; years 1997–2013, codes F84.0, F84.1, F84.5, F84.8, F84.9). Similar to an earlier study [3], we did not consider the timing of diagnosis meaningful and regarded children as having ASD from early age regardless of when the first diagnosis was registered. We did this because age of diagnosis is dependent on several factors, such as availability of services and the diagnostic routines and preferences of the time, which has changed between 1987 and 2013. Further, the severity of ASD also affects the age when caregivers seek help and diagnostic assessment is conducted. DSM-5 criteria are consistent with this reasoning, stating that symptoms of ASD must be present in childhood but might not necessarily be fully manifested until later life, when social demands exceed the individuals’ capabilities [13].

To ensure a complete picture of potential stoppage effects, we defined being exposed to ASD in families in several ways:

1. *Any before*—Any previously born child by the same mother had an ASD diagnosis.
2. *Immediately before*—The immediately previously born child having ASD diagnosis.
3. *Birth order specific*—For first- to second-born children we compared families where the first-born had ASD diagnosis to families where the first-born was not diagnosed with ASD. For second- to third-born, we calculated exposure as (a) only first-born having ASD diagnosis, (b) only second-born having ASD diagnosis, and (c) both first- and second-born having ASD diagnosis and compared to families without children with ASD diagnosis.

### Outcomes

#### Inter-pregnancy intervals

We calculated the time from a birth until next birth, or censoring, for births up until fifth child of a mother.

#### Number of subsequent children

We counted the number of children born to the same mother after each birth. To account for differing time of follow-up, we calculated time remaining until censored or age 55 for mothers for each first to fourth births. The choice of 55 as the upper limit was guided by the data, since almost no births were observed after this age.

### Covariates

As covariates (all categorical), we firstly included birth order, a confounder since the more siblings in a family the more likely that at least one of them has ASD, and if parents plan their family size, birth order will also affect the outcome. Secondly, we included birth period (1987–1991, 1992–1996, 1997–2001, 2002–2006, 2007–2011) to alleviate spurious associations due to changes in rates of ASD diagnoses (steeply increasing over time in current sample) and changes in average family sizes over time. Thirdly, maternal and paternal age were investigated, since they are associated with probability of ASD in offspring [14] and with likelihood of having more children. Fourthly, we included highest maternal education, gathered from the Longitudinal Database for Health Insurance and Labor Market Studies [15], as a proxy of socioeconomic status, as it may be associated with both risk for ASD and family size. Finally, we considered sex of offspring in index birth, since the risk for ASD differs between the sexes and that the sex composition of the siblings may affect parents’ decision to have another child.

## Statistical analyses

### Descriptive

For children with and without an ASD diagnosis, we summarized the data on individual level (for covariates sex, birth order, maternal and paternal age), family level (number of children, follow-up time, rate of childbearing, family size, maternal education level), and inter-pregnancy interval level (observed and number censored intervals, interval length). We calculated inverse Kaplan-Meier curves (i.e., 1 minus the survival function) for the cumulative proportion having another child according to the three exposure definitions.

### Inter-pregnancy intervals

We performed survival analyses using Cox proportional hazards regression, where event was defined as “having another child,” to analyze the potential stoppage effect in terms of delaying next child and/or not having another child. We analyzed the data for the different exposure definitions: crude, adjusted for one covariate at time, and then adjusted for all covariates. For analyses with exposure definitions (1) and (2), i.e., where birth orders were combined, all included children of a mother were considered, and adjustment for birth order was performed by stratified Cox regression to allow differing baseline hazards.

### Number of subsequent children

We used Poisson regression to analyze the childbearing rate after an index childbirth to examine the potential limiting of planned family size. The number of subsequent children was considered the outcome; the time left that a mother possibly could have children (i.e., until censored or 55 years of age) was included as an offset term in the analyses. We also split the follow-up time into different maternal age periods (according to levels in Table 1) and counted the number of children born within each period to allow different baseline rates of childbearing within different maternal ages. We analyzed the data for the different exposure definitions: crude, adjusted for one covariate at time, and then adjusted for all covariates.

**Table 1:** Descriptive information on the first four children born in analyzed families, on individual-, family-, and inter-pregnancy interval levels

Individual level descriptive information		Children with ASD diagnosis	Children without ASD diagnosis
Number of individuals (% of total)		26,842 (1.1)	2,476,592 (98.9)
		Number of individuals (column %)	Number of individuals (column %)
Sex	Male	19,081 (71.1)	1,268,115 (51.2)
	Female	7761 (28.9)	1,208,477 (48.8)
Birth order	1	15,383 (57.3)	1,254,634 (50.7)
	2	8414 (31.3)	889,411 (35.9)
	3	2472 (9.2)	270,100 (10.9)
	4	573 (2.1)	62,447 (2.5)
Maternal age	< 20	1084 (4.0)	74,574 (3.0)
	20–24	6173 (23.0)	476,466 (19.2)
	25–29	9292 (34.6)	852,599 (34.4)
	30–34	6912 (25.8)	730,188 (29.5)
	35–39	2868 (10.7)	292,915 (11.8)
	40–44	494 (1.8)	47,978 (1.9)
	≥ 45	19 (0.1)	1872 (0.1)
Paternal age	< 20	872 (3.2)	103,102 (4.2)
	20–24	3103 (11.6)	219,819 (8.9)
	25–29	7487 (27.9)	659,913 (26.6)
	30–34	7933 (29.6)	788,390 (31.8)
	35–39	4518 (16.8)	456,482 (18.4)
	40–44	1841 (6.9)	169,901 (6.9)
	45–49	731 (2.7)	54,048 (2.2)
	≥ 50	357 (1.3)	24,937 (1.0)
Family level descriptive information		Families with children with ASD diagnosis^a^	Families without children with ASD diagnosis^a^
Number of families (% of total)		25,489 (2.0)	1,244,528 (98.0)
Childbearing rate	Total number of children (% of total)^b^	61,842 (2.4)	2,480,416 (97.6)
	Mean family size	2.4	2.0
	Total years of follow-up	451,368.4	16,145,609.0
	Average years of follow-up per mother	17.6	13.0
	Rate (children/1000 years)	137.7	153.6
		Number of families (column %)	Number of families (column %)
Family size	1	3813 (15.0)	368,379 (29.6)
	2	11,873 (46.6)	613,380 (49.3)
	3	6714 (26.3)	202,838 (16.3)
	4	2118 (8.3)	43,233 (3.5)
	≥ 5	971 (3.8)	16,698 (1.3)
Highest maternal education level	Information not available	171 (0.7)	36,829 (3.0)
	Primary and lower secondary education less than 9 years	469 (1.8)	31,339 (2.5)
	Primary and lower secondary education 9 years	2386 (9.4)	81,628 (6.6)
	Upper secondary education 1–2 years	6581 (25.8)	221,040 (17.8)
	Upper secondary education 3 years	5466 (21.4)	296,444 (23.8)
	Post-secondary education less than 3 years	3867 (15.2)	182,915 (14.7)
	Post-secondary education 3 years or longer	6304 (24.7)	379,577 (30.5)
	Postgraduate education	245 (1.0)	14,756 (1.2)
Inter-pregnancy intervals Initial birth before January 1, 2012		Inter-pregnancy intervals with an immediately prior born child with ASD diagnosis	Inter-pregnancy intervals with an immediately prior born child without ASD diagnosis
Number of inter-pregnancy intervals (% of total)		26,831 (1.2)	2,269,306 (98.8)
	Inter-pregnancy intervals that ended with a birth (% within each group)	16,156 (60.2)	1,230,071 (54.2)
		Number of inter-pregnancy intervals (% per birth interval and group)	Number of inter-pregnancy intervals (% per birth interval and group)
Inter-pregnancy intervals	Observed 1st to 2nd	11,636 (75.7)	882,951 (76.1)
	Censored 1st to 2nd	3742 (24.3)	277,066 (23.9)
	Observed 2nd to 3rd	3451 (41.0)	268,023 (33.1)
	Censored 2nd to 3rd	4962 (59.0)	542,798 (66.9)
	Observed 3rd to 4th	844 (34.2)	61,803 (25.4)
	Censored 3rd to 4th	1623 (65.8)	181,271 (74.6)
	Observed 4th to 5th	225 (39.3)	17,294 (31.2)
	Censored 4th to 5th	348 (60.7)	38,100 (68.8)
		Mean time (standard deviation)	Mean time (standard deviation)
	Observed 1st to 2nd	3.5 (2.5)	3.3 (2.1)
	Censored 1st to 2nd	15.3 (6.7)	10.9 (7.8)
	Observed 2nd to 3rd	4.4 (2.9)	4.3 (2.8)
	Censored 2nd to 3rd	15.1 (5.6)	11.9 (6.7)
	Observed 3rd to 4th	4.2 (3.0)	3.9 (2.7)
	Censored 3rd to 4th	13.8 (5.0)	10.7 (5.9)
	Observed 4th to 5th	3.5 (2.4)	3.5 (2.4)
	Censored 4th to 5th	12.3 (4.5)	9.2 (5.1)

*ASD* autism spectrum disorder

^a^Families with children with ASD diagnosis refers to families where any of the four first-born children receive an ASD diagnosis; families without children with ASD diagnosis are the families where no one of the four first-born children receive any ASD diagnosis

^b^All children included, not limited to first- to fourth-born

### Sensitivity analysis

#### Birth order, third- to fourth-born

For third- to fourth-born inter-pregnancy interval, we calculated inverse Kaplan-Meier curves where we compared families without children with ASD diagnosis with families where (a) only first-born had ASD, (b) only second-born had ASD, (c) only third-born had ASD, and (d) at least two of first- to third-born having ASD.

#### Birth cohort specific associations

To investigate potential bias arising from differing length of follow-up—for instance, bias due to too short follow-up to ascertain diagnosis in the offspring—we performed analyses for inter-pregnancy intervals stratified on birth year of first-born (in same categories as listed above).

All analyses were performed using R base functions [16] and library “survival” [17]. Precision of estimates are presented as 95% confidence intervals, adjusted using the sandwich estimator, to account for deviations from basic modeling assumptions (such as homoscedasticity), from the “sandwich” library [18].

We tested the proportional hazards assumption for the most inclusive “Any before”-exposure definition. The test showed an acceptable low deviation from proportionality; the correlation between the Schoenfeld residuals and Kaplan-Meier-transformed time was − 0.002 with a *p* value of 0.012 (an acceptable *p* value given the large sample [19]).

### Role of the funding source

The funders had no role in the design of the study; collection, analysis, and interpretation of the data; in writing the report; and decision to submit the manuscript for publication.

## Results

### Descriptive

In Table 1, descriptive information on the population is presented. Our analyzed cohort, the first four children born to a mother, comprises a total of 1,270,017 families with 2,503,434 children with a total of 26,842 (1.1%) children with ASD diagnosis. More males than females were diagnosed with ASD, and diagnosed children were more likely to be first-born. Moreover, children with ASD were generally born to younger parents. Families with children with ASD diagnosis had, on average, more children than those without (2.4 vs 2.0), and a longer follow-up (17.6 vs 13.0 years), but a lower mean rate of childbearing (137.7 vs 153.6 children per 1000 years). Further, mothers in families with children with ASD diagnosis had lower education level than mothers in families without. For inter-pregnancy intervals, the number of intervals ending with a birth (rather than being censored) was higher in families with children with ASD diagnosis (60.2%) than in families without (54.2%). This was also observed by birth order, except for first- to second-born where families without first-born with ASD diagnosis showed a higher rate of observed second births. Families with children with ASD diagnosis generally had longer inter-pregnancy intervals between two consecutive births, as well as from a birth until censoring.

**Fig. 1:**
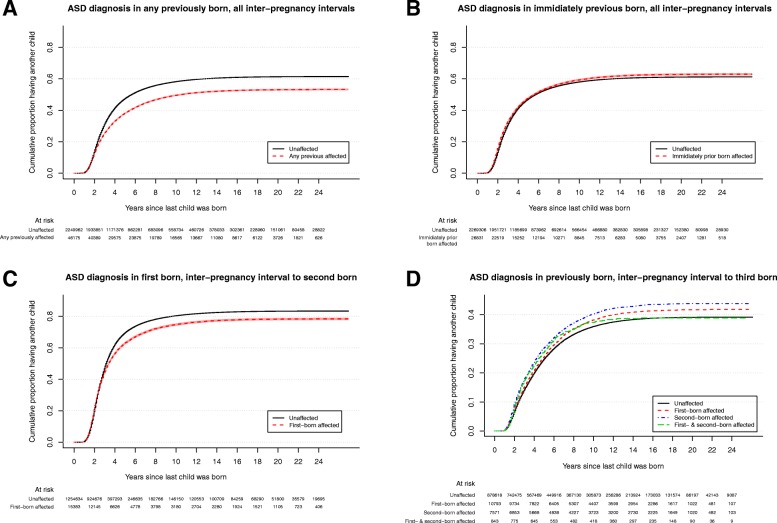
**a** Proportion having another child if any of the previous children received an ASD diagnosis. All inter-pregnancy intervals included. Estimates, 95% confidence intervals, and numbers at risk. **b** Proportion having another child if the immediately prior born child received an ASD diagnosis. All inter-pregnancy intervals included. Estimates, 95% confidence intervals, and numbers at risk. **c** Proportion having a second child if the first-born child receives an ASD diagnosis. Estimates, 95% confidence intervals, and numbers at risk. **d** Proportion having a third child if the first- and second-born children received ASD diagnoses. Estimates and numbers at risk (note: different scale on *y*-axis; in Additional file 1: Figure S1, this plot is presented with 95% confidence intervals)

Figure 1a show a pattern indicating a potential stoppage effect, collapsed over all childbirths; families with prior born children with ASD diagnosis had less subsequent children. In Fig. 1b, the same graph is presented but considering if the immediately prior born child has ASD, whereafter the pattern supportive for stoppage completely disappears. Further, while families with children with ASD diagnosis show a *lower* likelihood of getting a second child if the first-born has ASD diagnosis (Fig. 1c), families with two children show a *higher* likelihood to get a third child if any of the two have an ASD diagnosis (Fig. 1d).

### Inter-pregnancy intervals

In Table 2, all Cox analyses for inter-pregnancy intervals are presented. The crude HRs, using no covariates, reflected in Fig. 1, suggested that mothers of ASD diagnosed children waited longer until having another child and/or were less likely to have another child compared to mothers of children without ASD (hazard ratio [HR], 0.79; 95% confidence interval [CI], 0.78–0.80). Among the considered covariates, birth order attenuated this effect most (HR, 0.97; 95% CI, 0.96–0.98). When all covariates were adjusted for, the association was non-existing (HR, 1.00; 95% CI, 0.99–1.02), indicating that the result was due to differences in considered covariates between families with or without children with ASD diagnosis. The HRs were above unity when only immediately previous child was considered as exposure (HR, 1.05; 95% CI, 1.03–1.07) but changed to below unity after covariate adjustment (HR, 0.97; 95% CI, 0.95–0.98).

**Table 2:** Analyses of inter-pregnancy intervals, Cox proportional hazards regression of time from one birth to next

	Hazard ratio (95% confidence interval)					
	All birth orders combined		Birth order specific			
			First to second	Second to third		
	ASD in any previously born	ASD in immediately previously born	ASD in first-born	ASD in first-born	ASD in second-born	ASD in both first- and second-born
Crude						
	0.79 (0.78–0.80)	1.05 (1.03–1.07)	0.87 (0.86–0.89)	1.08 (1.05–1.11)	1.17 (1.13–1.22)	1.05 (0.93–1.17)
Adjusted						
Birth order^a^	0.97 (0.96–0.99)	0.94 (0.92–0.96)	NA	NA	NA	NA
Maternal age	0.82 (0.81–0.83)	1.01 (1.00–1.03)	0.88 (0.87–0.90)	1.09 (1.05–1.12)	1.11 (1.08–1.15)	1.06 (0.95–1.19)
Birth period	0.80 (0.79–0.81)	1.04 (1.02–1.05)	0.88 (0.86–0.89)	1.09 (1.05–1.12)	1.17 (1.13–1.21)	1.06 (0.95–1.19)
Sex	0.78 (0.77–0.79)	1.05 (1.03–1.06)	0.87 (0.85–0.89)	1.08 (1.05–1.11)	1.17 (1.13–1.21)	1.04 (0.93–1.17)
Paternal age	0.81 (0.8–0.82)	1.03 (1.01–1.04)	0.88 (0.86–0.89)	1.09 (1.06–1.12)	1.14 (1.10–1.18)	1.08 (0.96–1.20)
Maternal education	0.79 (0.78–0.81)	1.06 (1.05–1.08)	0.88 (0.86–0.90)	1.10 (1.06–1.13)	1.18 (1.14–1.22)	1.05 (0.94–1.17)
All of above^a^	1.00 (0.99–1.02)	0.97 (0.95–0.98)	0.90 (0.88–0.91)	1.12 (1.08–1.15)	1.16 (1.12–1.20)	1.11 (0.99–1.24)

*NA* not applicable, *ASD* autism spectrum disorder

^a^Birth order adjustment by stratification (stratified Cox), where applicable

For first to second pregnancy interval, the unadjusted HR was 0.87 (95% CI, 0.86–0.89) and remained lower than unity after covariate adjustment (HR, 0.90; 95% CI, 0.88–0.91). Conversely, for second to third pregnancy intervals, the HRs were well above unity and remained so after covariate adjustment (HR range 1.05 to 1.17), suggesting that families with ASD diagnosed children had shorter intervals between children and/or were more likely to have another child.

### Number of subsequent children

In Table 3, all Poisson regression analyses of childbearing rates are presented. As observed in the descriptive data, the crude rate of childbearing in families with any previous child having an ASD diagnosis was lower than in families without children with ASD diagnosis, rate ratio (RR), 0.85 (95% CI, 0.84–0.86), meaning that families with children with ASD diagnosis had 15% less subsequent children. Adjustment for birth order had the largest effect on this estimate (RR, 0.97; 95% CI, 0.96–0.98). The effect was reversed when all covariates were adjusted for and when baseline birth rates were modeled to be different for different maternal ages (RR, 1.04; 95% CI, 1.03–1.05). The results were similar for the immediately previous born having ASD, but of smaller magnitude.

**Table 3:** Analysis of number of subsequent children, Poisson regression analysis of rates as number of children per follow-up time

	Rate ratio (95% confidence interval)					
	All birth orders combined		Birth order specific			
			Rate of children after first-born	Rate of children after second-born		
	ASD in any previously born	ASD in immediately previously born	ASD in first-born	ASD in first-born	ASD in second-born	ASD in both first- and second-born
Crude						
	0.85 (0.84–0.86)	0.98 (0.96–0.99)	0.90 (0.89–0.91)	1.07 (1.04–1.10)	1.08 (1.05–1.11)	1.07 (0.97–1.18)
Adjusted						
Birth order	0.97 (0.96–0.98)	0.94 (0.93–0.95)	NA	NA	NA	NA
Maternal age^a^	0.91 (0.9–0.92)	1.00 (0.99–1.01)	0.94 (0.93–0.95)	1.09 (1.06–1.12)	1.11 (1.08–1.14)	1.10 (1.00–1.20)
Birth period	0.87 (0.87–0.88)	1.04 (1.02–1.05)	0.95 (0.94–0.97)	1.07 (1.05–1.11)	1.14 (1.11–1.18)	1.11 (1.00–1.22)
Sex	0.85 (0.84–0.86)	0.98 (0.96–0.99)	0.90 (0.89–0.91)	1.07 (1.04–1.10)	1.08 (1.05–1.11)	1.07 (0.97–1.18)
Paternal age	0.88 (0.87–0.89)	0.97 (0.96–0.99)	0.91 (0.90–0.92)	1.08 (1.05–1.11)	1.06 (1.03–1.09)	1.09 (0.99–1.20)
Maternal education	0.87 (0.87–0.88)	1.00 (0.99–1.01)	0.92 (0.91–0.93)	1.11 (1.08–1.14)	1.11 (1.08–1.14)	1.10 (1.00–1.21)
All of above^a^	1.04 (1.03–1.05)	1.02 (1.01–1.03)	0.98 (0.96–0.99)	1.11 (1.08–1.14)	1.15 (1.12–1.18)	1.14 (1.04–1.24)

Poisson model with number of following children as outcome and time of follow-up as offset term. Mothers are assumed to not be able to have children after age 54

*NA* not applicable, *ASD* autism spectrum disorder

^a^Analysis adjusted for different baseline rates of children in different maternal age intervals (< 20, 20–24, 25–29, 30–34, 35–39, 40–44, 45–54).

When analyzing numbers of subsequent children after first-born, comparing ASD diagnosed with undiagnosed, we found a limiting effect both in the crude (RR, 0.90; 95% CI, 0.89–0.92) and fully adjusted model (RR, 0.98; 95% CI, 0.96–0.99), similarly to the analysis of inter-pregnancy intervals. By contrast, the analysis of number of subsequent children after second-born showed that families with ASD diagnosed children had higher rates of childbearing, with and without covariate adjustments (RR range 1.06 to 1.15).

### Sensitivity analysis

#### Birth order, third- to fourth-born

The inverse Kaplan-Meier curves for third- to fourth-born indicated higher number of children in families with children with ASD diagnosis (Additional file 1: Figures S2 and S3).

#### Birth cohort specific associations

When analyzing inter-pregnancy intervals in families stratified on the first-born child’s birth years, the result remained similar (Additional file 1: Table S1). The birth order specific estimates had CIs that all contained the estimate from the main analyses. In all birth periods, the result from combined birth orders had CIs containing 1 or the full CI was above 1, except the last birth period (with shortest follow-up) with HR and CI below 1. However, potential problems with bias due to lack of information on diagnosis should be largest in the birth cohort with shortest follow-up.

## Discussion

This study investigated the hypothesis that parents having a child diagnosed with ASD change their reproductive behaviors towards having fewer subsequent children—commonly referred to as reproductive stoppage. We used two variables capturing stoppage effects, inter-pregnancy interval (time to next child), and number of subsequent children in a population sample of 2,521,103 children in 1,270,017 families. Our results did not support the hypothesis of a universal stoppage effect in families with children with ASD diagnosis, but results depended on birth orders. The group of parents whose first-born had an ASD diagnosis tended to be less likely to have another child and had smaller families. However, among those who did have a second child, and among those who had a child with ASD diagnosis of later birth order, the overall family size was larger than in families without children with ASD diagnosis. Notably, when analyzing these effects combined over birth order (and other covariates), they tended to balance out for inter-pregnancy intervals (Table 2) and to be in the direction of more children in families with children with ASD diagnosis for number of subsequent children (Table 3).

Although our results did not confirm previous research regarding universal stoppage [3, 4], it concurs with a Danish study for first to second birth [4]. They, however, did not consider a combined effect over birth orders; therefore, we cannot compare the combined effects directly between the cohorts. Furthermore, the results from a Californian study [3] differ in magnitude of estimates from ours and in direction/significance of estimates in adjusted analyses. Both previous large studies only analyzed time-to-next-child; we are thus unable to compare our results regarding number of subsequent children directly.

The observed differences may be due to societal differences between Scandinavian countries and California. Healthcare in Sweden is largely tax-funded, a system that ensures everyone has equal access to healthcare services, as well as to other welfare and support systems (e.g., parental leave). In the USA, families with children with ASD diagnosis might suffer financially more than in Sweden. This might explain why a stoppage effect is present in the Californian study but not in the present study. Further studies are needed in order to investigate if the healthcare system affects reproductive behavior. Alternatively, the difference could be due to ascertainment differences; the present study is based on population-based health registers, minimizing the risk of selection bias, while the Californian study was based on a non-random sample from a target population rather than total population, which may explain some of the difference in results.

The experience of having a child with ASD may differ across families. Some parents may experience more psychological strain of rearing a child with ASD and therefore choose to not have subsequent children (i.e., the lower rate of second births in these families). Relatedly, the ASD phenotype is highly heterogeneous with some individuals requiring almost constant support from early childhood, while, for others, the ASD symptomatology does not manifest itself until late childhood, and then often in milder forms. Thus, the severity of the ASD phenotype may affect the choice to conceive another child. Additionally, parental characteristics expressed when becoming a parent and in the parent-child relationship vary and, therefore, the parents’ psychological perception of having a child with ASD may differ. Hence, it could be that it is not the ASD phenotype per se that causes the observed associations but, rather, the parental experience. Possibly, these two effects interact with regard to parental decision on future childbearing. Thus, future research may investigate the psychological ramifications and potential differences of experience for parents of having a child with ASD diagnosis.

Finally, our results indicate that, in family studies of ASD, proper attention to covariates, particularly birth order, may be sufficient to alleviate potential biases due to differences in childbearing in families with children with ASD diagnosis compared to those without, at least when familial effects are not considered to be birth order specific.

## Limitations

The results of this study needs to be viewed in light of certain limitations. We based our ASD definition on the National Patient Register, where we are likely to face an under-detection of true cases in the population, since not all cases are diagnosed in clinical practice and reported to the register. However, the under-reporting is likely to be small since ASD is today apparently rather consequently diagnosed in Sweden, as indicated by relatively high national prevalence rates from a comparative international view [20, 21], and the majority of individuals with ASD will receive care by specialist care who report to the National Patient Register. We assumed ASD present prior to it being observed in the registries, which potentially introduce bias. However, a sensitivity analysis based on families with different birth years of first-born child yielded results which did not deviate notably from the overall results. Finally, following the current dominating umbrella concept of ASD, we did not differentiate between the included ASD sub-diagnoses; it is possible that reproductive patterns can be dependent on ASD subtypes and the severity and composition of ASD phenotypes and comorbidities.

The main strengths of this study are its large sample of a non-selected population and the prospectively collected data, as well as the several ways the exposure and outcome were conceptualized. Importantly, Sweden has equal health access for its inhabitants making bias due to systematic differences in likelihood of being diagnosed unlikely. Further, analyses of an entire cohort with prospectively collected data, as in the present study, minimizes risk of non-representability of analyzed sample in terms of comparisons of families with and without children with ASD diagnosis.

## Conclusion

This population-based cohort study of more than 2,500,000 individuals does not support the hypothesis of a universal reproductive stoppage effect in ASD families, when birth order and other factors are considered. Proper attention to birth order may alleviate potential bias in familial aggregation studies of ASD.

## Supplementary information

**Additional file 1: Figures S1–S3** and **Table S1.** Supplementary figures and tables.

## Data Availability

The datasets analyzed during the current study are not publicly available due to Swedish Law, but are available from the register holders (Statistics Sweden; Swedish National Board of Health and Welfare) on request, after ethical vetting by an Ethical Review Board.

## Abbreviations

ASD: Autism spectrum disorder
CI: Confidence interval
HR: Hazard ratio
RR: Rate ratio
