# Supplementary material for: Reproductive stoppage in autism spectrum disorder in a population of 2.5 million individuals
Source: Mol Autism. 2019 Dec 11;10:45. doi: 10.1186/s13229-019-0300-6 (PMC6907273; doi:10.1186/s13229-019-0300-6)
Supplement: Supplementary file 1 — Additional file 1: Figures S1–S3 and Table S1. Supplementary figures and tables. [file 13229_2019_300_MOESM1_ESM.docx]

**Additional file 1**

**Reproductive stoppage in autism spectrum disorder in a population of 2.5 million individuals**

Ralf Kuja-Halkola,^1^ Henrik Larsson,^1,2^ Sebastian Lundström,^3^ Sven Sandin,^1,4,5^ Azadeh Chizarifard,^6^ Sven Bölte,^7,8,9^ Paul Lichtenstein,^1^ EmmaFrans^1^

^1^ Department of Medical Epidemiology and Biostatistics, Karolinska Institutet, Stockholm, Sweden

^2^ School of Medical Sciences, Örebro University, Örebro, Sweden

^3^ Gillberg Neuropsychiatry Centre; Centre for Ethics, Law and Mental Health, University of Gothenburg, Gothenburg, Sweden

^4^ Department of Psychiatry, Icahn School of Medicine at Mount Sinai, New York, USA

^5^ Seaver Autism Center for Research and Treatment at Mount Sinai, New York, USA

^6^ Department of Statistics, Stockholm University, Stockholm, Sweden

^7^ Center of Neurodevelopmental Disorders (KIND), Centre for Psychiatry Research; Department of Women’s and Children’s Health, Karolinska Institutet & Stockholm Health Care Services, Region Stockholm, Stockholm, Sweden

^8^ Child and Adolescent Psychiatry, Stockholm Health Care Services, Region Stockholm, Stockholm, Sweden

^9^ Curtin Autism Research Group, School of Occupational Therapy, Social Work and Speech Pathology, Curtin University, Perth, WA, Australia

**Corresponding author:** Ralf Kuja-Halkola, Department of Medical Epidemiology and Biostatistics, Karolinska Institutet, PO Box 281, SE-171 77 Stockholm, Sweden. Email: [ralf.kuja-halkola@ki.se](mailto:ralf.kuja-halkola@ki.se).

**Figure S1:** Proportion having a third child if the first- and second born children received ASD diagnoses. Estimates, 95% confidence intervals and numbers at risk

**
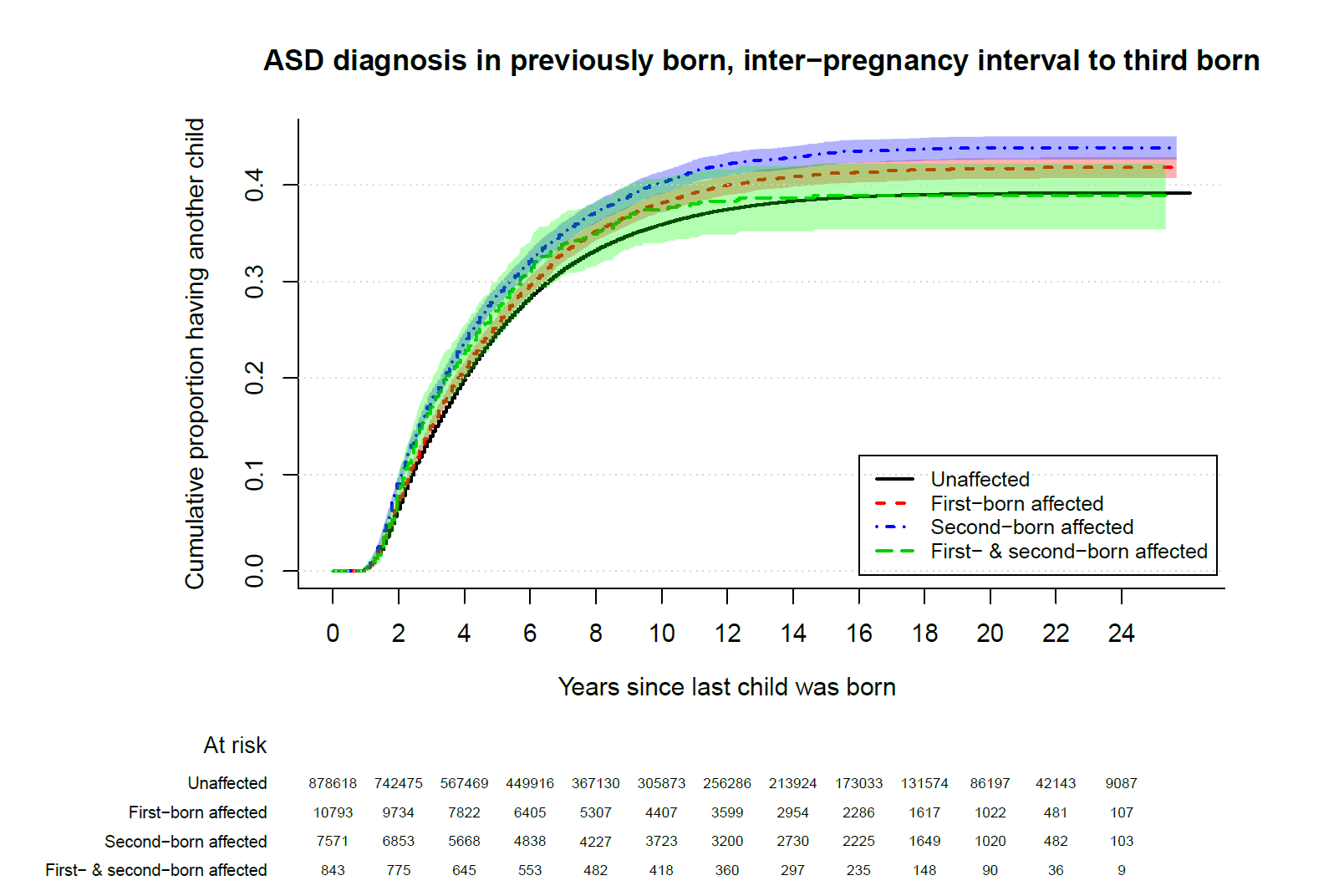
**

**Figure S2:** Proportion having a fourth child grouped into ASD-diagnoses in first- to third-born. Estimates and numbers at risk.

**
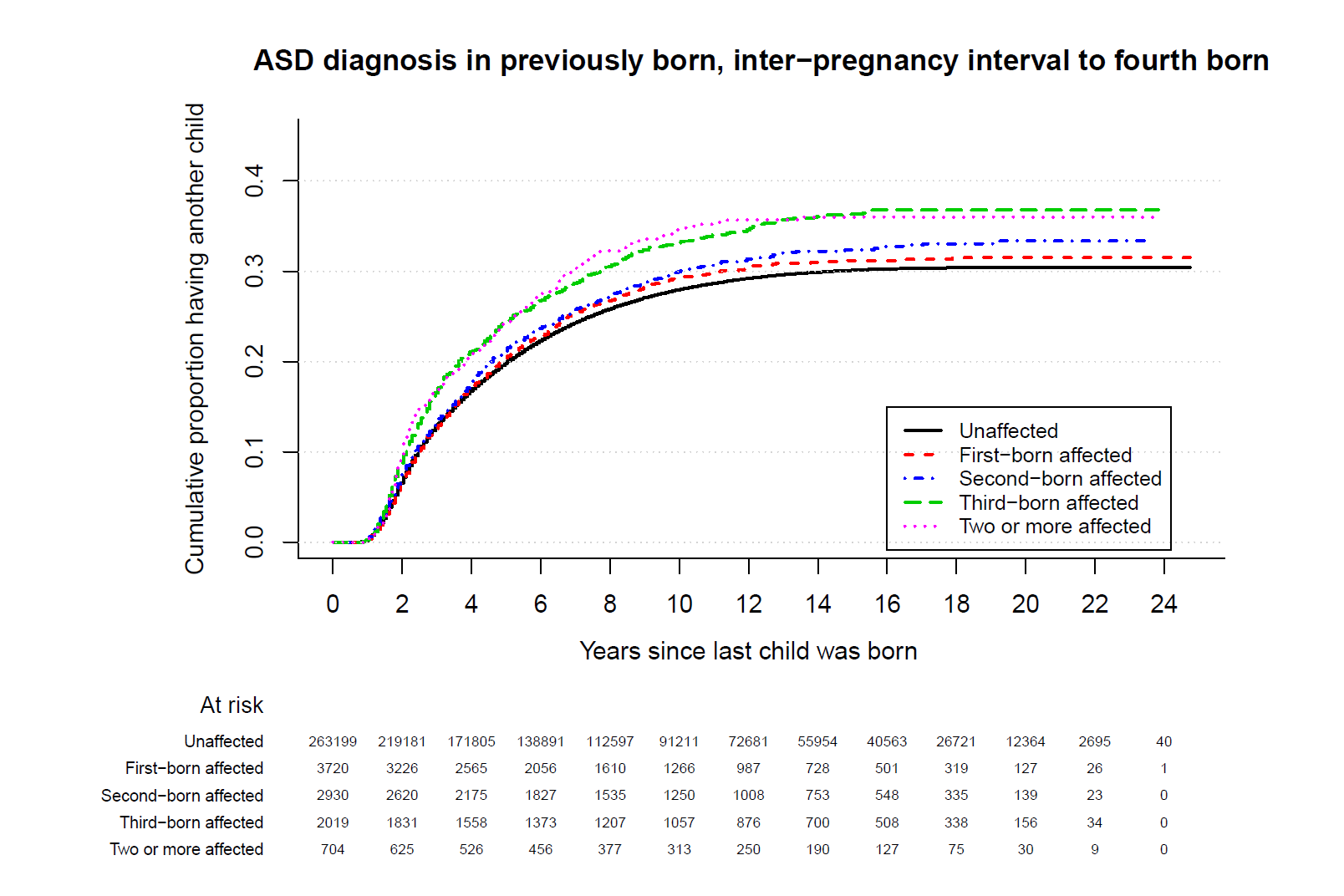
**

**Figure S3:** Proportion having a fourth child grouped into ASD-diagnoses in first- to third-born. Estimates, 95% confidence intervals and numbers at risk.

**
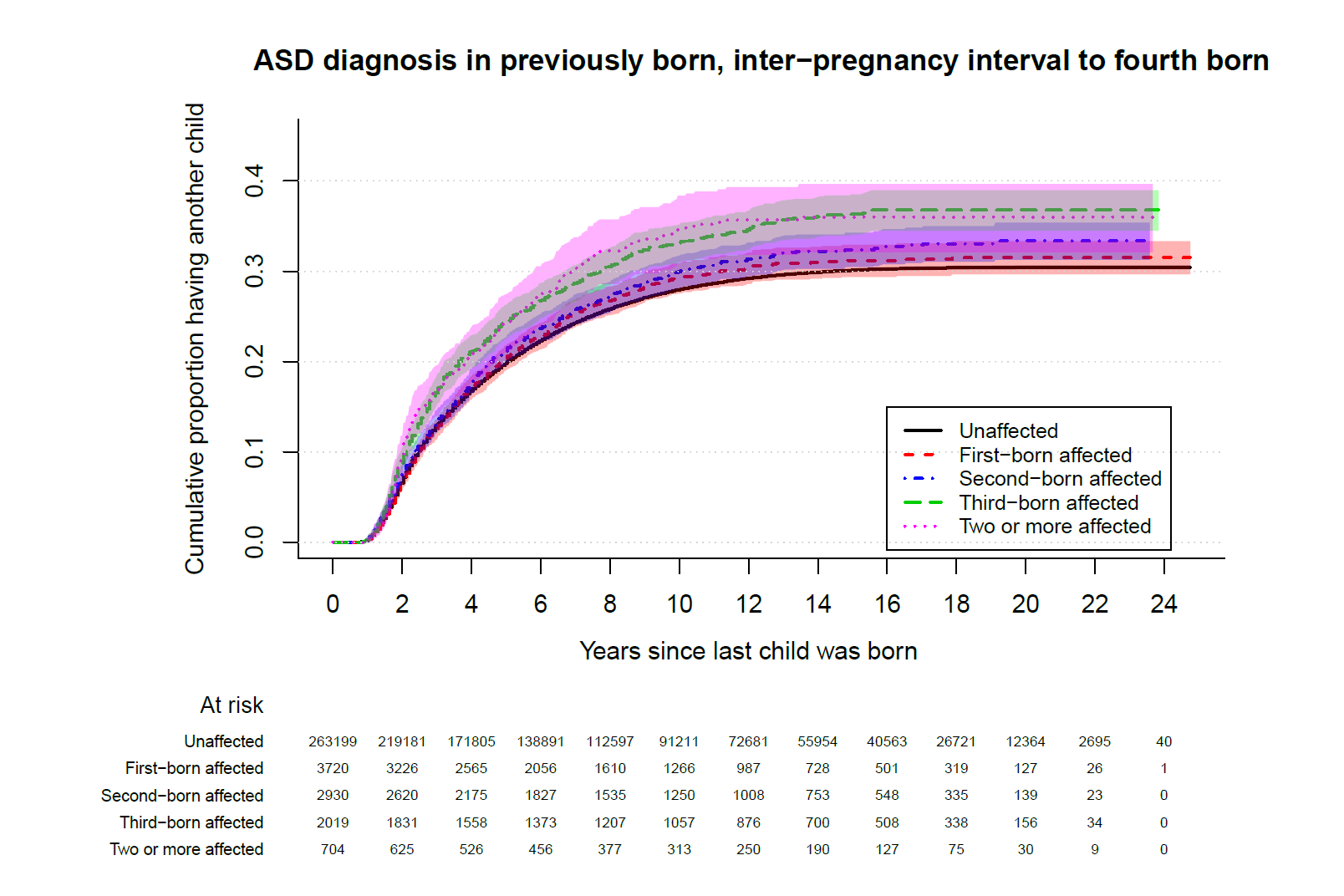
**

**Table S1.** Analyses of inter-pregnancy intervals, Cox proportional hazards regression of time from one birth to next.

|  |  | **Hazard Ratio (95% confidence interval)** | | | | | |
| --- | --- | --- | --- | --- | --- | --- | --- |
|  |  | **All birth orders combined** | | **Birth order specific** | | | |
|  |  |  |  | **First to second** | **Second to third** | | |
|  | **Year of mother’s first pregnancy** | ASD in any previously born | ASD in immediately previously born | ASD in first born | ASD in first born | ASD in second born | ASD in both first- and second born |
| **Crude** | All^b^ | 0.79 (0.78-0.80) | 1.05 (1.03-1.07) | 0.87 (0.86-0.89) | 1.08 (1.05-1.11) | 1.17 (1.13-1.22) | 1.05 (0.93-1.17) |
|  | 1987-1991 | 0.76 (0.75-0.78) | 0.99 (0.96-1.01) | 0.87 (0.84-0.90) | 1.10 (1.04-1.16) | 1.13 (1.07-1.19) | 0.94 (0.75-1.17) |
|  | 1992-1996 | 0.83 (0.81-0.85) | 1.10 (1.07-1.13) | 0.88 (0.85-0.91) | 1.10 (1.04-1.16) | 1.16 (1.09-1.24) | 1.07 (0.89-1.27) |
|  | 1997-2001 | 0.86 (0.84-0.88) | 1.17 (1.13-1.21) | 0.87 (0.84-0.90) | 1.09 (1.03-1.17) | 1.19 (1.10-1.29) | 1.05 (0.82-1.35) |
|  | 2002-2006 | 0.88 (0.85-0.91) | 1.24 (1.19-1.29) | 0.90 (0.86-0.95) | 1.04 (0.95-1.13) | 1.34 (1.19-1.51) | 1.39 (1.01-1.91) |
|  | 2007-2011 | 0.77 (0.72-0.83) | 0.96 (0.88-1.04) | 0.84 (0.78-0.92) | 0.90 (0.71-1.14) | 1.39 (0.98-1.97) | 0.96 (0.31-2.98) |
| **Adjusted**^a^ | All^b^ | 1.00 (0.99-1.02) | 0.97 (0.95-0.98) | 0.90 (0.88-0.91) | 1.12 (1.08-1.15) | 1.16 (1.12-1.20) | 1.11 (0.99-1.24) |
|  | 1987-1991 | 1.03 (1.01-1.06) | 1.00 (0.97-1.03) | 0.90 (0.86-0.93) | 1.15 (1.09-1.22) | 1.14 (1.08-1.21) | 0.99 (0.79-1.24) |
|  | 1992-1996 | 1.01 (0.99-1.04) | 0.98 (0.95-1.00) | 0.90 (0.87-0.93) | 1.15 (1.08-1.21) | 1.14 (1.07-1.21) | 1.16 (0.97-1.37) |
|  | 1997-2001 | 0.99 (0.96-1.02) | 0.95 (0.91-0.98) | 0.90 (0.86-0.93) | 1.09 (1.02-1.16) | 1.16 (1.07-1.26) | 1.04 (0.81-1.34) |
|  | 2002-2006 | 0.98 (0.95-1.02) | 0.97 (0.92-1.01) | 0.92 (0.88-0.96) | 1.03 (0.95-1.13) | 1.25 (1.11-1.41) | 1.39 (1.00-1.94) |
|  | 2007-2011 | 0.91 (0.84-0.98) | 0.91 (0.83-0.98) | 0.89 (0.82-0.97) | 0.90 (0.71-1.14) | 1.36 (0.95-1.95) | 0.94 (0.32-2.75) |

Notes: ASD, autism spectrum disorder.
^a^ Adjusted for birth order (by stratified Cox), maternal age, birth period, sex, paternal age, maternal education, and birth period, where applicable.

^b^ Same result is in **Table 2** in main article.
